# NLAS-multi: A multilingual corpus of automatically generated Natural Language Argumentation Schemes

**DOI:** 10.1016/j.dib.2024.111087

**Published:** 2024-10-29

**Authors:** Ramon Ruiz-Dolz, Joaquin Taverner, John Lawrence, Chris Reed

**Affiliations:** a Centre for Argument Technology, University of Dundee, Dundee DD1 4HN, United Kingdom; b VRAIN, Universitat Politècnica de València, València 46022, Spain

**Keywords:** Argument mining, Natural language generation, Computational argumentation

## Abstract

Some of the major limitations identified in the areas of argument mining, argument generation, and natural language argument analysis are related to the complexity of annotating argumentatively rich data, the limited size of these corpora, and the constraints that represent the different languages and domains in which these data is annotated. To address these limitations, in this paper we present the following two contributions: an effective methodology for the automatic generation of natural language arguments in different topics and languages, and the largest publicly available corpus of Natural Language Argumentation Schemes available to date.

Specifications Table

**Table:** 

Subject	Computer Science: Artificial Intelligence.
Specific subject area	Computational Argumentation and Natural Language Processing.
Type of data	*Please list your type(s) of data and data formats. Delete any description from the lists that does not apply. If your data type is not featured, please manually add it.* JSON file Analyzed, annotated, validated
Data collection	The data was retrieved from interactions with Large Language Models, backed by concepts from argumentation theory, and validated by expert annotators*.*
Data source location	Not applicable*:*
Data accessibility	Repository name: Zenodo - NLAS-multi: A Multilingual Corpus of Automatically Generated Natural Language Argumentation Schemes Data identification number (DOI): 10.5281/zenodo.8364002 Direct URL to data: https://doi.org/10.5281/zenodo.8364002
Related research article	none*.*

## 1 Value of the Data

• The NLAS-multi corpus represents the largest publicly available dataset of natural language arguments, comprising 1893 arguments in English and 1917 in Spanish. Making it one of the largest multilingual argumentation datasets.
• The NLAS-multi corpus includes 20 different argumentation schemes, covering the widest set of Walton's argumentation schemes in an annotated dataset, and enabling state-of-the-art Natural Language Processing models to learn linguistic features from stereotyped patterns of argumentative reasoning.
• The results from the creation of the NLAS-multi corpus reveal that generative Large Language Models are powerful tools to create arguments following well-defined structures, achieving an accuracy above 90 % after the two-phase process.
• The NLAS-multi corpus allows new research to investigate several tasks that remained unexplored due to the lack of large-enough data, such as automatic scheme identification or enthymeme reconstruction.

## 2 Background

The dataset presented in this paper is theoretically grounded on two main concepts: Large Language Models [1,2], and Walton's argumentation schemes [3]. The argumentation schemes proposed and defined by Walton provide a mature model of argument representation in which the natural language and the logical structure of the argument can be partially dissociated. The argumentation scheme model can be understood as an intermediate representation between formal and informal representations of human argumentation. While research in formal computational argumentation dispenses with natural language [4,5], informal argumentation research is mainly based in natural language instances of human argumentation [[6], [7], [8]]. However, argumentation schemes combine aspects from formal logic and abstract representations with natural language to define different types of argumentative reasoning patterns. Let us consider the Argument from Position to Know scheme as an example, Walton defines this argument as:

Major Premise: Source s is in position to know about things in a certain subject domain f containing proposition p.
Minor Premise: s asserts that p is true.
Conclusion: p is true.

An argumentation scheme thus provides a set of abstract variables that can be replaced with natural language text, together with the connections between these variables required to make it each specific argumentation scheme.

## 3 Data Description

In this paper we present NLAS-multi, a compilation of 3810 textbook-like natural language arguments in two languages, English and Spanish, representing the largest available corpora consisting of Walton's argumentation schemes. The arguments included in NLAS-multi have been generated automatically and validated by human annotators, making sure that they follow the right structure defined in [3]. Our corpus contains arguments instantiating 20 different argumentation schemes, in 50 different topics, and two stances (i.e., in favour and against the topic). The 20 argumentation schemes include: Argument From Analogy (AFAN), Argument From Best Explanation (AFBE), Argument From Cause to Effect (AFCE), Argument From Expert Opinion (AFEO), Argument From Example (AFEX), Argument From Ignorance (AFIG), Argument From Position to Know (AFPK), Argument From Popular Opinion (AFPO), Argument From Popular Practice (AFPP), Argument From Precedent (AFPR), Argument From Established Rule (AFRL), Argument From Sunk Costs (AFSC), Argument From Sign (AFSI), Argument From Threat (AFTH), Argument From Verbal Classification (AFVC), Argument From Waste (AFWS), Argument From Witness Testimony (AFWT), Direct ad Hominem (DAH), Inconsistent Commitment (IC), Slippery Slope (SS).

Table 1 describes the inferences, words, and NLAS generated for the 20 argumentative schemes in the NLAS-multi corpus per argumentation scheme. To understand the distribution of the NLAS-multi corpus, it is essential to emphasise that it has been generated from intentionally developed atomic arguments, with conflicting positions (i.e., arguments for and against for each argumentative scheme and each topic) and adapted to different argumentative schemes that include predefined premises and conclusions. This directly affects the number of inferences, NLAS per topic, and conflicts, as it is be discussed below.

**Table 1:** Description of the number of inferences, words, and arguments in the corpus per argumentation scheme.

English					Spanish			
	Inferences	Words	Arguments		Inferences	Words	Arguments	
			In Favour	Against			In Favour	Against
Total	3,949	118,493	941	952	4,015	135,023	974	943
Mean	197.5	5,924.7	47.1	47.6	200.8	6,751.2	48.7	47.2
Sd	49.4	1,787.1	6.5	3.4	54.5	1,993.4	3.2	7.1

The NLAS-multi corpus consists of a total of 253,516 words distributed in 118,493 words in English and 135,023 words in Spanish. This makes our corpus one of the most extensive currently available. Comparing it with some of the largest publicly available argumentation corpora in the literature, we find that our corpus is significantly larger than the VivesDebate corpus [9] with 139,756 words, and slightly smaller than the QT30 corpus [10] with 279,996 words. In terms of inferences, our corpus has a total of 7964 (3949 in English and 4015 in Spanish). This places our corpus below ViveDebate with 12,653 and above QT30 with 5205 inferences. However, it is important to note that both VivesDebate and QT30 were extracted from videos or radio programs with real and open debates. In contrast, as previously mentioned, the NLAS-multi corpus consists of textbook-like atomic arguments modelled from argumentative schemes with a predetermined structure (premises and conclusions). Thus, the number of inferences in our corpus is determined by the number of premises defined in each argument scheme (i.e., from each premise an inference to the argument statement is deduced), unlike VivesDebate and QT30, which allowed a free format for argumentation. Therefore, we can find that arguments such as Argument from Example or Direct Ad Hominem which, having only one premise, present a lower number of inferences (97 and 100 respectively in English and 100 in both cases in Spanish), while arguments such as Argument from Best Explanation or Argument from Witness Testimony, which have three premises, have a higher number of inferences (300 both in English and, 297 and 300 in Spanish respectively). Finally, regarding the stance, in English, approximately 49.7 % of the arguments are in favour, while 50.3 % are against. In Spanish, the proportion is 50.8 % of arguments in favour and 49.2 % against. In both languages most of the arguments have around 50 samples of each type.

Table 2 describes of the number of inferences, conflicts, and arguments for the 50 topics included in the corpus, per topic. The variance in both English and Spanish is relatively low, indicating that most of the topics have a number close to 80 inferences. In the case of English, the minimum is 73 inferences for the topic ‘Physical appearance for personal success’, while the maximum is 84 for topics such as ‘Mandatory vaccination in pandemic’ or ‘Renewable energy’. Similarly, in Spanish, the minimum is 74 inferences for topics such as ‘Freedom of speech’ or ‘Climate change’, while the maximum is 84 for topics such as ‘Internet censorship’ or ‘Surrogacy’. Furthermore, the NLAS-multi corpus contains a total of 23,781 conflict relations. Thanks to the strategy employed in the development of the corpus, which involves the use of conflicting positions for each argumentative schema and each topic, our corpus has a significantly higher number of conflicts compared to previous corpora in the literature. For example, the QT30 corpus contains only 976 conflicts, while the VivesDebate corpus is limited to 1,558. Finally, the distribution of NLAS in favour and against the 50 topics is close in both cases to 19, with standard deviations of 1 or less (the maximum value of 20 corresponds to the 20 argumentation schemes). The distribution of NLAS is relatively uniform, with the minimum being 16 for the topic ‘Physical appearance for personal success’ in English, and 15 for the topic ‘Climate change’ in Spanish.

**Table 2:** Description of number of inferences, conflicts, and arguments per topic.

English					Spanish			
	Inferences	Conflicts	Arguments		Inferences	Conflicts	Arguments	
			In Favour	Against			In Favour	Against
Total	3,949	11,626	941	952	4,015	12,155	974	943
Mean	79.0	232.5	18.8	19.0	80.3	243.1	19.5	18.9
Sd	3.2	16.2	1.0	0.9	3.0	17.6	0.7	1.0

## 4 Experimental Design, Materials and Methods

In this section, we describe the process used to build the corpus through the generation of Natural Language Argumentation Scheme (NLAS) using the APIs of GPT-3.5-turbo and GPT-4. Each argument was instantiated with 50 different topics including claims referring to Euthanasia, Mandatory vaccination in pandemic, Climate change, and UFO existence. In addition, the arguments were generated with two stances: in favor and against. This resulted in the generation of 2000 arguments (20 schemes x 50 topics x 2 stances) for two languages: English and Spanish.

**Fig. 1:**
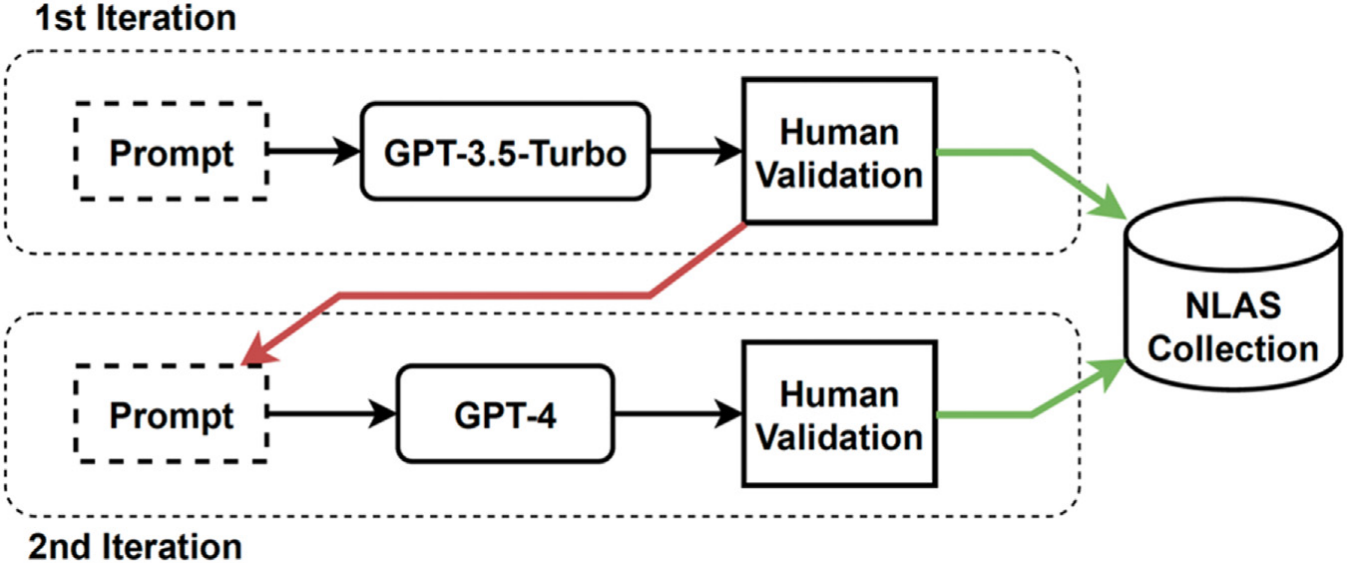
Automatic generation of NLAS.

Fig. 1 illustrates the procedure followed to generate the NLAS-multi corpus. This procedure is divided into two iterations. In a first iteration, we generated 2000 arguments for each language (English and Spanish) using GPT-3.5-turbo. These arguments were evaluated by five expert annotators: four for English and two for Spanish (one annotator worked with both languages). During this evaluation, the experts considered only the adequacy of the argument generated in relation to the pattern of each argumentation scheme, the topic, and the stance and annotate these arguments as valid or not valid. Arguments that were classified as not valid (i.e., arguments that did not adhere to the argumentative structure, topic, or stance) were discarded.

In a second iteration, GPT-4 was used to re-generate the arguments that were discarded in the first iteration using the same prompt design. Expert annotators evaluated the generated arguments using the same criteria as in the evaluation of the first iteration. Finally, arguments that were classified as suitable by the expert evaluators in both iterations were used to generate the NLAS-multi corpus. Note that, our objective is to obtain a labelled corpus, i.e., we are not evaluating the GPT-3.5-turbo and GPT-4 performance. Therefore, this two-step methodology is justified by the reduction of the economic, temporal, and computational cost that using GPT-3.5-turbo provides to us.

### 4.1 Subsection: Natural Language Generation

To generate the arguments, we followed a communication strategy with the APIs of GPT-3.5-turbo and GPT-4, applying prompting techniques. With these techniques, we aimed to maintain the original structure of each argument scheme proposed by Walton. To achieve this, the general structure of our prompt comprised three main parts: the argument type specification, the argument pattern, and the output format.

For the argument type specification, we request that the system generates a specific argument type (e.g., Argument from Position to Know) by defining the stance (i.e., in favor or against) and the topic (e.g., euthanasia). Following these guidelines, an example of the argument type specification would be: `Provide a position to know argument in favor of euthanasia'. Next, we attached the template of the argument scheme pattern to be instantiated. The scheme was presented in accordance with the specifications proposed by Walton for each type of argument.

Finally, in the prompt, we specified the desired output format to be provided in response to the request. We requested that the output should be in JSON format, maintaining the structure of the argumentation schema provided. This way, our final prompt was structured as follows:

*“Provide an {scheme} argument {stance} on the topic of topic following the argument pattern:*

*{argument_description}*

*You must answer in a .json format following the structure of the argumentation scheme.”*

Where variables such as {scheme}, {stance}, {topic}, or {argument_description} were adapted each time covering our 20 argumentation schemes, 50 topics, and 2 stances to obtain the variety and richness described above.

The design of the prompt is based on the work proposed in [11,12]. We conducted an initial round of exploratory experiments to test the design of the communication with the API through prompts. The objective was to establish prompts to generate arguments on different topics and stances, while complying with the structure of the argumentation schemes. However, considering some issues in the formal logic consistency of argumentation schemes identified in previous research [13], this initial round of experiments produced unsatisfactory outcomes. In some argumentation schemes, the system was unable to identify the variables that needed to be instantiated. Taking the example of Argument from Position to Know, the language model often fails to recognise the variable s as a candidate for being instantiated, resulting in the return of the argument structure without instantiating that variable. To address this problem, we adopted the approach of using the structure ‘[variable]’ to denote the variables as discussed in [14]. In an initial attempt, we introduced square brackets to the variables within Walton's original scheme (e.g., ‘source s’ was revised to ‘source [s]’). While this adjustment significantly enhanced the system's capability to instantiate arguments, issues persisted when instantiating some variables. These problems arose due to the system's inability to discern which element to instantiate within each variable. Therefore, we opted to redefine the specification of the argument schemes to make the variables more explicit while maintaining the consistence of the argumentation scheme. Thus, the scheme for the Argument from Position to Know was described as:

Major Premise: [Someone] is in position to know about things in a certain subject domain [Domain] containing proposition [A],
Minor Premise: [Someone] asserts that [A] is true,
Conclusion: [A] is true

This way, the generative language model was able to identify that the variable [Someone] in the Major Premise should be modified by the name of a person (or the name of a group or collective) and use that name in the Minor Premise.

Once the structure of the prompt was determined, we generated the 2000 arguments for English and Spanish using the APIs of GPT-3.5-turbo and GPT-4 according to the process described above. The following is an example of the argument generated for the Argument from Position to Know scheme on the topic of euthanasia and with the stance in favor using GPT-3.5-turbo:

Major Premise: Medical professionals (such as doctors and nurses) are in position to know about the treatment options available for terminally ill patients.
Minor Premise: Many medical professionals argue that euthanasia is a humane option for terminally ill patients who are experiencing unbearable suffering and have little hope for recovery.
Conclusion: Euthanasia can be a morally justifiable option for terminally ill patients who are experiencing unbearable suffering and have little hope for recovery.

As can be seen in this example, GPT-3.5-turbo was able to modify the syntactic structure of the argument, enriching the content, without affecting the semantics or the structure of the argument scheme.

### 4.2 Subsection: Human Validation

After the natural language generation of arguments, two human validation steps were carried out. For that purpose, we hired five professional linguists with specialised training in argumentation theory, well versed in the analysis and annotation of arguments. In addition, we provided them with the formal definitions of the argumentation schemes included in our experiments to ensure a consistent application. Each annotator made their evaluations individually, drawing on their training and familiarity with the argumentation schemes. Their task was to check whether a NLAS was valid or not considering its argumentation scheme structure, its topic, and its stance. Only in the case of an argument following the expected argumentation scheme structure, arguing about the expected topic, and aligned with the expected stance was considered as valid. If any of these variables was violated, the argument was annotated as non-valid and rejected from our collection. It is important to emphasise that aspects such as the soundness, persuasiveness, or the strength of an argument were not considered in our annotation. Therefore, some arguments that might not be perceived as solid by humans were still labelled as valid if they respected all the variables described as relevant. Regarding the five annotators, four were native English speakers, who annotated the arguments in English, while two worked on the arguments in Spanish. Among the two annotators of the Spanish corpus, one was a native Spanish speaker and the other was a bilingual annotator who was fluent in both English and Spanish and worked in both language sets.

**Fig. 2:**
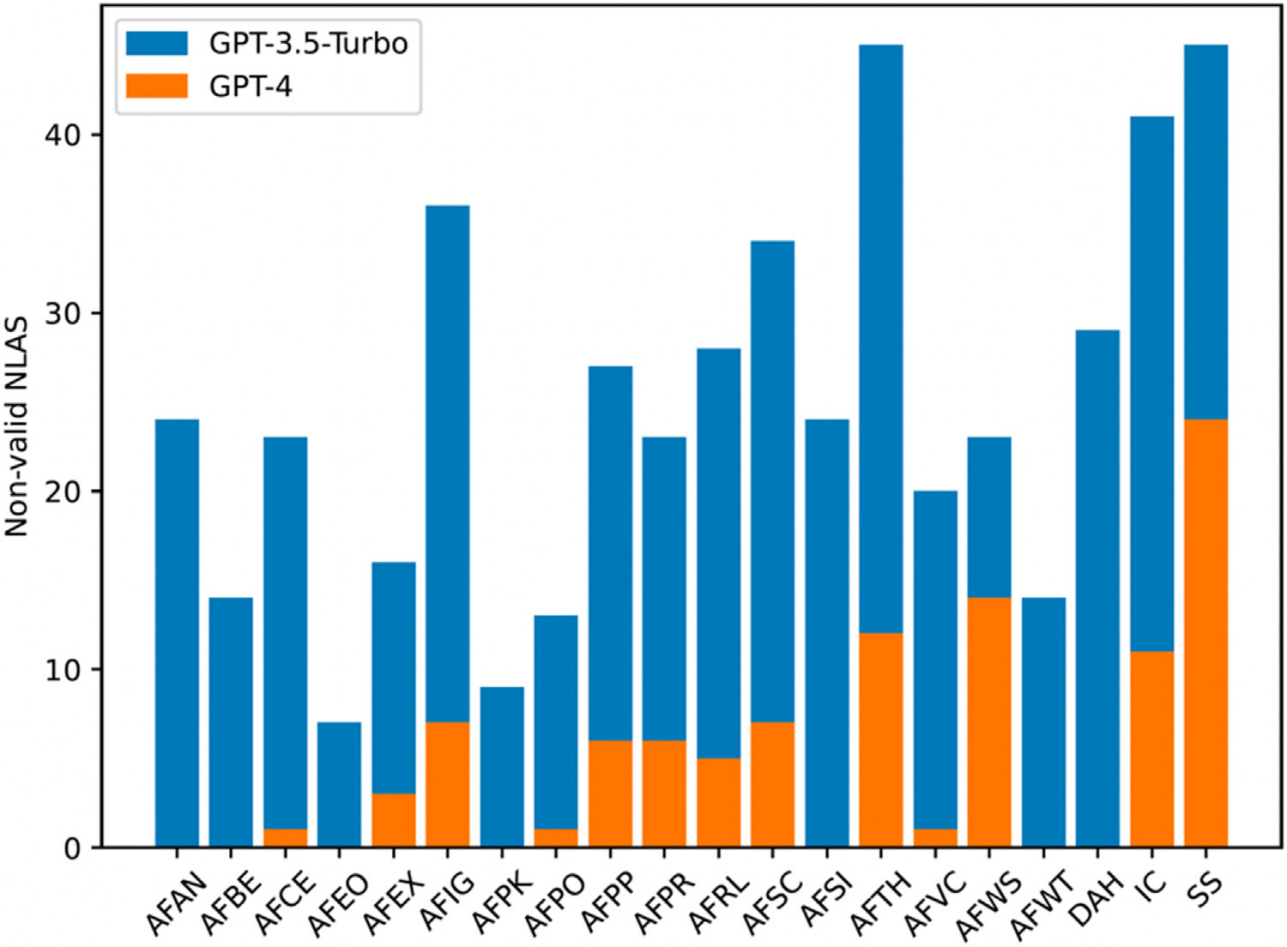
Non-valid English NLAS.

**Fig. 3:**
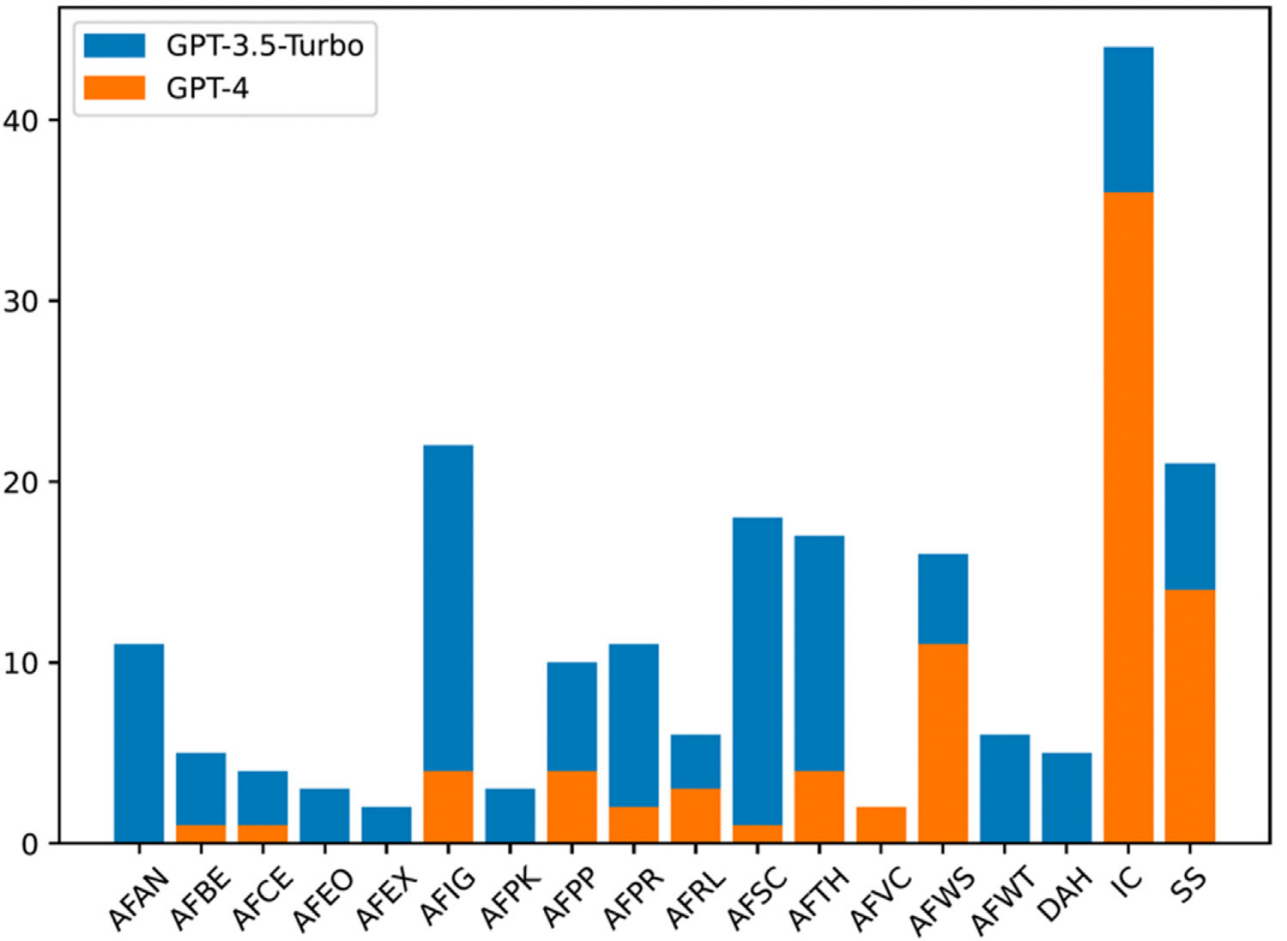
Non-valid Spanish NLAS.

In the first human validation step, we analysed the NLAS produced in the first natural language generation iteration with GPT-3.5-turbo. Therefore, in this first step, 2000 natural language arguments were manually annotated in English, and another 2000 in Spanish. Regarding the English part we obtained 1496 valid and 504 non-valid NLAS, meaning that with GPT-3.5-turbo we were able to achieve an initial accuracy of 74.8 %. From this first validation, we observed that the error distribution was not uniform, some argumentation schemes were more challenging than others during the natural language generation part of the process (see Fig. 2, blue bars). Regarding the Spanish part, we obtained 1794 valid and 206 non-valid NLAS, achieving an initial accuracy of an 89.7 %. In the Spanish part, we observed a significant smaller amount of non-valid NLAS than in English, and the error distribution was mainly dominated the argument from inconsistent commitment (see Fig. 3, blue bars). The error distribution of the other argumentation schemes was significantly lower than in English.

In the second human validation step, we analysed the NLAS produced in the second natural language generation iteration with GPT-4. Compared to the first step, in this second step we only re-generated the arguments that were annotated as non-valid in the first iteration. Therefore, 504 NLAS were manually annotated in English and 206 in Spanish in the second human validation step. As for the English part, in the end of this second step we obtained 397 more valid NLAS and 107 non-valid arguments. Compared to the first iteration, GPT-4 was able to improve the performance by generating correctly a 78.8 % of the natural language arguments that GPT-3.5-turbo failed to generate as intended. This was a big improvement, allowing us to correctly generate a total of 1893 NLAS out of the 2000 prompted arguments (i.e., a 94.7 %) following our proposed methodology. The errors identified in this second validation step, however, were also not distributed uniformly between the different types of argumentation schemes (see Fig. 2, orange bars). As for the the second iteration with the Spanish part, we obtained 123 valid and 83 non-valid NLAS, achieving an accuracy of 60 % with GPT-4. This improvement allowed us to generate a total of 1917 valid NLAS, a 95.8 % of the prompted argumentation schemes. Again, the error in this second validation step was focused on the inconsistent commitment argumentation scheme (see Fig. 3, orange bars).

To validate our annotation, we carried out two Inter Annotator Agreement (IAA) studies containing a 10 % subset of the total number of samples, one for each language (i.e., 200 NLAS per language). Regarding the IAA in English, we report a 0.65 Cohen's Kappa [15], meaning that there was a substantial agreement between the four annotators. On the other hand, we report a 0.22 Cohen's Kappa in the Spanish annotation, meaning that our annotators reached only a fair agreement. The agreement in the Spanish part of the corpus was significantly lower than the one observed in the English part, however, we observed that the total number of disagreements was lower than in the English study (i.e., 21 disagreements in Spanish versus 23 in English). This variation can be attributed to the significantly low number of non-valid arguments annotated in the Spanish part compared to the English part of the corpus. A disagreement in a non-valid argument represents a higher ratio to the absolute number of invalid arguments compared to the English part, and this has a direct effect to the Cohen's Kappa score.

## Limitations

The NLAS-multi corpus, although being one of the larger and richer argument corpora in terms of argumentation schemes, consists of compiled schemes that are textbook-like arguments containing complete reasoning structures. Humans, however, do not typically use such structures when arguing, making use of enthymemes, namely arguments where one or multiple premises have been intentionally omitted. It remains future work, therefore, to explore ways of leveraging the argumentation schemes included in NLAS-multi to make the models trained on this data effective for natural argumentation analysis.

## Ethics Statement

We have read the ethical requirements for publication in Data in Brief and the current work does not involve human subjects, animal experiments, or any data collected from social media platforms*.*

## CRediT authorship contribution statement

**Ramon Ruiz-Dolz:** Conceptualization, Methodology, Software, Visualization, Project administration, Writing – original draft. **Joaquin Taverner:** Conceptualization, Methodology, Formal analysis, Writing – original draft. **John Lawrence:** Writing – review & editing, Funding acquisition, Supervision. **Chris Reed:** Writing – review & editing, Funding acquisition, Supervision.

## Data Availability

ZenodoNLAS-multi: A Multilingual Corpus of Automatically Generated Natural Language Argumentation Schemes (Original data).
